# The future of zoonotic risk prediction

**DOI:** 10.1098/rstb.2020.0358

**Published:** 2021-11-08

**Authors:** Colin J. Carlson, Maxwell J. Farrell, Zoe Grange, Barbara A. Han, Nardus Mollentze, Alexandra L. Phelan, Angela L. Rasmussen, Gregory F. Albery, Bernard Bett, David M. Brett-Major, Lily E. Cohen, Tad Dallas, Evan A. Eskew, Anna C. Fagre, Kristian M. Forbes, Rory Gibb, Sam Halabi, Charlotte C. Hammer, Rebecca Katz, Jason Kindrachuk, Renata L. Muylaert, Felicia B. Nutter, Joseph Ogola, Kevin J. Olival, Michelle Rourke, Sadie J. Ryan, Noam Ross, Stephanie N. Seifert, Tarja Sironen, Claire J. Standley, Kishana Taylor, Marietjie Venter, Paul W. Webala

**Affiliations:** ^1^ Center for Global Health Science and Security, Georgetown University Medical Center, Washington, DC 20007, USA; ^2^ Department of Microbiology and Immunology, Georgetown University Medical Center, Washington, DC 20007, USA; ^3^ Department of Ecology and Evolutionary Biology, University of Toronto, Toronto, Ontario, Canada; ^4^ Public Health Scotland, Glasgow G2 6QE, UK; ^5^ Cary Institute of Ecosystem Studies, Millbrook, NY 12545, USA; ^6^ Medical Research Council, University of Glasgow Centre for Virus Research, Glasgow G61 1QH, UK; ^7^ Institute of Biodiversity, Animal Health and Comparative Medicine, College of Medical, Veterinary and Life Sciences, University of Glasgow, Glasgow G12 8QQ, UK; ^8^ O'Neill Institute for National and Global Health Law, Georgetown University Law Center, Washington, DC 20001, USA; ^9^ Department of Biology, Georgetown University, Washington, DC 20007, USA; ^10^ Animal and Human Health Program, International Livestock Research Institute, PO Box 30709-00100, Nairobi, Kenya; ^11^ Department of Epidemiology, College of Public Health, University of Nebraska Medical Center, Omaha, NE, USA; ^12^ Icahn School of Medicine at Mount Sinai, New York, NY, USA; ^13^ Department of Biological Sciences, Louisiana State University, Baton Rouge, LA 70806, USA; ^14^ Department of Biology, Pacific Lutheran University, Tacoma, WA, USA; ^15^ Department of Microbiology, Immunology, and Pathology, College of Veterinary Medicine and Biomedical Sciences, Colorado State University, Fort Collins, CO, USA; ^16^ Department of Biological Sciences, University of Arkansas, Fayetteville, AR 72701, USA; ^17^ Centre on Climate Change and Planetary Health, London School of Hygiene and Tropical Medicine, London, UK; ^18^ Centre for Mathematical Modelling of Infectious Diseases, London School of Hygiene and Tropical Medicine, London, UK; ^19^ Centre for the Study of Existential Risk, University of Cambridge, Cambridge, UK; ^20^ Department of Medical Microbiology and Infectious Diseases, University of Manitoba, Winnipeg, Manitoba, Canada R3E 0J9; ^21^ Molecular Epidemiology and Public Health Laboratory, Hopkirk Research Institute, Massey University, Palmerston North, New Zealand; ^22^ Department of Infectious Disease and Global Health, Cummings School of Veterinary Medicine, Tufts University, North Grafton, MA 01536, USA; ^23^ Department of Public Health and Community Medicine, School of Medicine, Tufts University, Boston, MA 02111, USA; ^24^ University of Nairobi, Nairobi, Kenya; ^25^ EcoHealth Alliance, New York, NY 10018, USA; ^26^ Law Futures Centre, Griffith Law School, Griffith University, Nathan, Queensland 4111, Australia; ^27^ Department of Geography and Emerging Pathogens Institute, University of Florida, Gainesville, FL, USA; ^28^ School of Life Sciences, University of KwaZulu-Natal, Durban, South Africa; ^29^ Paul G. Allen School for Global Health, Washington State University, Pullman, WA, USA; ^30^ Department of Virology, University of Helsinki, Helsinki, Finland; ^31^ Department of Veterinary Biosciences, University of Helsinki, Helsinki, Finland; ^32^ Department of Chemical Engineering, Carnegie Mellon University, Pittsburgh, PA 15213, USA; ^33^ Zoonotic Arbo and Respiratory Virus Program, Centre for Viral Zoonoses, Department of Medical Virology, University of Pretoria, Pretoria, South Africa; ^34^ Department of Forestry and Wildlife Management, Maasai Mara University, Narok 20500, Kenya

**Keywords:** zoonotic risk, epidemic risk, access and benefit sharing, machine learning, global health, viral ecology

## Abstract

In the light of the urgency raised by the COVID-19 pandemic, global investment in wildlife virology is likely to increase, and new surveillance programmes will identify hundreds of novel viruses that might someday pose a threat to humans. To support the extensive task of laboratory characterization, scientists may increasingly rely on data-driven rubrics or machine learning models that learn from known zoonoses to identify which animal pathogens could someday pose a threat to global health. We synthesize the findings of an interdisciplinary workshop on zoonotic risk technologies to answer the following questions. What are the prerequisites, in terms of open data, equity and interdisciplinary collaboration, to the development and application of those tools? What effect could the technology have on global health? Who would control that technology, who would have access to it and who would benefit from it? Would it improve pandemic prevention? Could it create new challenges?

This article is part of the theme issue ‘Infectious disease macroecology: parasite diversity and dynamics across the globe’.

## 1. Introduction

**Figure 1.**
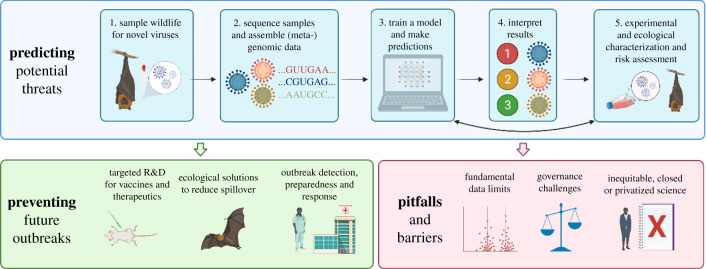
Zoonotic risk technology can be part of a broader scientific pipeline that connects viral discovery and wildlife disease surveillance to the development of biomedical and ecological solutions to predict, prevent and prepare for future outbreaks. Created with BioRender.com. (Online version in colour.)

After the COVID-19 pandemic ends—or even before [1]—the world will face another emergence of a heretofore-unknown epidemic or pandemic threat, which will most likely be a novel zoonotic virus. This is less a testament to the state of global health, and more a basic consequence of arithmetic: as many as one in every five known mammalian viruses has the ability to make the jump into human populations, and only an estimated 1% of mammal viruses are currently known to science [2,3]. For example, a whole constellation of distinct SARS-related coronaviruses circulate in bats and in China and Southeast Asia [4,5], and at least two-thirds of reservoirs might still be unidentified [6]. However, even the most intensively studied viruses and well-sampled hosts can harbour undiscovered diversity: influenza A viruses are perhaps the most widely agreed upon future pandemic threat [7–9], but novel strains emerging through reassortment in wildlife and livestock are often only noted once they reach or cross the animal–human interface. Despite the urgency of research on zoonotic emergence, the diversity and rapid evolution of viruses poses a problem of scale for actionable science (figure 1).

The next zoonotic threat might be unfamiliar to virologists, but more likely than not, it will bear at least some similarity to previous counterparts. A handful of viral clades make the zoonotic jump most often, and are more likely to continue spreading within human populations [10–12]. As a result, novel zoonotic epidemics often harken back to previous outbreaks: severe acute respiratory syndrome coronavirus 2 (SARS-CoV-2) shares 76% of its genome with SARS-CoV, and much of its pathology [13]; the emergence of HIV-1 group M in the 1920s was followed by a dozen more spillovers of similar primate viruses, including the progenitor of HIV-2 [14–16]; and the emergence of filoviruses with Marburg virus in 1967 was followed almost a decade later by the first *Sudan ebolavirus* and *Zaire ebolavirus* outbreaks, both in 1976 [17].

Though these instances are anecdotal and few in number, similarities between emergence events across time and space point to the widely accepted idea that while individual outbreaks are idiosyncratic and (as standalone stochastic events) unpredictable, they often follow predictable patterns, which might constitute the raw materials for a zoonotic risk assessment procedure—defining, for example, the virus species, conditions or locations with a greater risk of causing or experiencing these events [18,19]. For example, a 2017 study, which aimed to predict risk factors for future coronavirus emergence, used a regression model to show that wildlife markets predicted higher coronavirus positivity rates in bats; used another model to estimate hundreds of coronaviruses might still be undiscovered and used a mapping approach to predict most undiscovered sarbecoviruses would be found in southern China and southeast Asia [20]. These predictions have been reasonably prescient, given the likely origins of SARS-CoV-2 in the southeast Asian peninsula through wildlife farming or trade 3 years later. Another study used simple models of viral sharing networks to predict that ferret badgers might have been a likely bridge host in the emergence of SARS-CoV-2 a year before the species was flagged by the World Health Organization origins investigation [6]. Though these approaches might not allow scientists to predict exactly where and when the next outbreak will begin, they allow a different kind of prediction, one focused on exploring and explaining biological possibility and socioecological risk factors with an eye towards future threats.

As zoonotic viruses and their non-human animal (hereafter animal) hosts become better characterized, a growing library of virological data is becoming increasingly available and accessible to the scientific community, putting this risk assessment procedure within reach for the first time. This is increasingly possible with the growing application of machine learning to risk assessment problems. Zoonotic origins are often described as a sequential process, in which pathogens must pass through a series of biological, ecological and social filters that would otherwise prevent their emergence [21,22]. At each of these steps, machine learning has been successfully and reliably applied to predict the animal origins of a novel zoonosis [23,24], the potential hosts of undiscovered zoonoses [6,25], the ecological and anthropogenic risk factors for zoonotic spillover [26,27], the ability of novel viruses to infect humans [28] and their ability to transmit onwards in human populations [10,29]. Such models have also been used to predict the severity of disease [30], and may be extended to predict mortality in the future [29,31]. These methods have been particularly useful when they can harness the genomic signatures of host adaptation and compatibility [23,32], as for many viruses, this may be the only available data [33].

Here, we focus on a subset of this emerging set of methods, which we term *zoonotic risk technology* and define as an informatic system, statistical model or artificial intelligence that identifies at least one of two viral traits we term *zoonotic potential* (which we define as the ability of an animal virus to infect a human host) and *epidemic potential* (the ability of a zoonotic infection to cause disease and transmit onwards in human populations). In calling these tools ‘zoonotic risk technology’, we aim to encompass a wider range of systems than just predictive models (e.g. informatic systems like databases that report risk factors for emergence based on expert opinion) and that could extend beyond them (e.g. the physical machines that can store these tools or would allow their deployment in field settings). More importantly, by considering these tools as a kind of emerging technology, we aim not to imply anything about the scientific validity or value of the tools, but instead to focus attention on their user base, implementation and effects on broader human systems. This set of approaches has a necessarily narrowly defined scope, which does not encompass every component of ‘risk’. For example, machine learning algorithms can also be applied to predict zoonotic isolates of bacteria [34,35], and similar models can be applied to identify potential wildlife reservoirs or arthropod vectors of zoonoses [25,36,37]. Similarly, spatio-temporal patterns of viral dynamics in livestock and wildlife reservoirs are a critical missing piece in many spillover risk assessments [38–40]. After a transmissible pathogen reaches human hosts, yet another set of virological, social, economic and political factors determine whether a spillover event becomes an epidemic or pandemic [41–44]. However, we focus on the narrowly defined idea of zoonotic risk technology as a way to operationalize a specific set of existing approaches to facilitate the identification of viruses with zoonotic potential, and to interrogate the potential value of these technologies to global health.

To facilitate discussion on these topics, we held a one-day digital workshop (the ‘Verena Forum on Zoonotic Risk Technology’) at the Georgetown University Center for Global Health Science and Security in January 2021. This setting allowed scientists to present cutting-edge computational and laboratory approaches, and to discuss potential applications or challenges with global health practitioners, with a focus on equity concerns in data sharing and technology deployment. Here, we report a brief synthesis of our findings.

Zoonotic risk technologies are no longer hypothetical, and are rapidly emerging as practical, concrete applications of scientific knowledge. These tools are part of the broader predictive toolkit in viral ecology, and like other kinds of predictive models, they are imperfect. Here, we identify three major barriers to actionable science that researchers must consider further:

(i) Technologies will have the most value to global health if they are treated as part of the process of characterizing risk, rather than the singular endpoint. Additional work is required to validate predictions, such as laboratory investigations, but may be expensive at-scale and potentially politically sensitive.
(ii) Academic publishing alone is insufficient to enable the deployment of tools in surveillance programmes or rapid outbreak response scenarios; user-friendly, open-source tools must be coupled with global capacity building in risk analyses and mitigation.
(iii) The development and application of zoonotic risk technology, and the sharing of data to enable these processes, are likely to engage critical issues such as ownership, equity and governance; these issues are considered central in global health, but relevant scholarship may not currently interface with existing research on zoonotic risk prediction.

We explore each of these issues in depth here, and discuss possible avenues for interdisciplinary work that might help overcome these barriers, identify conditions precedent to their use and flag potential limitations.

## 2. How zoonotic risk technology works

At its core, zoonotic risk technology exploits the assumption that viruses with undetected zoonotic potential are more similar to known zoonoses than to non-zoonotic viruses. Early efforts have focused on identifying coarse traits that are common among known zoonoses, such as origins in particular host clades [11,45,46] or a broad host range [47–49]. These approaches are useful for identifying common profiles of what a zoonosis ‘looks like’—e.g. a vector-borne single-stranded RNA virus with a broad host range including primates—that can be generalized across animal viruses. One of the only examples of zoonotic risk technology available for public use, the SpillOver viral risk ranking platform, uses this approach to rank 887 viruses based on 31 risk factors [50]. These approaches benefit from generality and interpretability, but can be limited by data availability; for example, host range is rarely characterized in wildlife viruses until they are a known threat to human health, and may suffer from biases in study effort or surveillance [51]. Moreover, trait-based assessments may be limited by apparent contradictions in simple patterns. For example, genome size correlates positively with zoonotic risk [52] but has been reported as having contradictory effects on transmissibility [10,12]; replication in the cytoplasm similarly predicts zoonotic potential [11,53,54], but also predicts reduced transmissibility [10]. Each of these has an idiosyncratic effect on risk assessment, but approaches that gather as many lines of evidence as possible can aim to minimize the influence of any given feature to an overall picture of risk.

Genomic data increasingly offer an alternate avenue for predictive work. Genomes are inherently high-dimensional data, encoding meaningful information about microbiology and immunology, and are often the first aspect of a novel virus to be characterized, months or years before its ecology. A simple model might be trained on the nucleotide similarity of viruses compared to known zoonotic threats, while a more advanced one might also include similarity in genomic composition biases [23,28]. This approach has worked well for the parallel problem of inferring viral origins using genomic features that encode coevolutionary signals of host adaptation. For example, CpG dinucleotide composition can be used to identify vertebrate viruses [55,56], exploiting a viral adaptation that matches genomic composition to the vertebrate genome in order to evade innate immune responses searching for non-self-genetic material [57,58]. These patterns are rare and poorly understood today [59], but the subject of significant interest. For zoonotic risk, a model is likely to identify some combination of broadly transferrable coevolutionary adaptations that allow a virus to cross species barriers more readily within a broad group (e.g. primates, or vertebrates), and random genomic patterns that happen to increase their odds of successful infection of human hosts (which they may never have encountered in their evolutionary history). For example, including similarity of viral genomes to human housekeeping genes and interferon-stimulated genes appears to measurably improve the prediction of zoonotic potential [28].

Over time, genomic approaches are likely to move beyond similarity, and start identifying de novo predictors of viral compatibility with human cells. A mechanism-agnostic model may simply collapse genomes into hundreds of computational features, identify a small handful of significant predictors and generalize these patterns—but can only do so successfully with sufficient data. For example, massive clearinghouses of genomic sequences such as GISAID and the NIAID Influenza Research Database have enabled a number of models that accurately classify the zoonotic potential of influenza strains down to the protein level [60,61]. Decomposing viral genomes, and identifying the regions most relevant to zoonotic emergence, can open new avenues for advanced modelling that go beyond pattern recognition. For example, researchers have developed a number of structural simulations to explore binding affinity between the spike protein of SARS-CoV-2 and ACE2 receptors in animal and human cells [62,63], and structural modelling can be paired with other trait data to better predict the capacity for various mammal species to transmit SARS-CoV-2 [64]. Similar approaches could be used to identify the zoonotic potential of other viruses for which surface protein structure and receptor use has been characterized [65]. These kinds of approaches are ultimately limited by the comparability of different structures in both host and pathogen genomes, and may be most predictive when comparing hosts or pathogens at lower taxonomic levels (i.e. viral strain up to viral genus or family).

No matter how sophisticated these approaches become, they all face the fundamental task of overcoming data limitations [51]. Only a few hundred zoonotic viruses are known—a sample size that is fundamentally limiting for inference, even when treated as the positives in a sample of a few thousand wildlife viruses. Many groups are chronically understudied, even after zoonotic viruses are discovered (e.g. the genus *Tibrovirus* remains poorly characterized despite the 2009 discovery of Bas-Congo virus and the 2015 discoveries of Ekpoma virus 1 and 2), creating bias in training datasets that will usually lead models to underestimate the risk posed by unfamiliar pathogens. Models that rely on similarity alone will particularly struggle with these data sources, while those that correctly identify underlying mechanisms (e.g. viral matching to host genomic composition) will perform better out-of-sample, though distinguishing the two can be challenging. Similarly, the genomes of many viruses are only partially characterized; models can train on specific regions of the genome, even though targets available from sequencing data may not be those with the greatest biological relevance (e.g. a recent model trained on betacoronavirus RdRp sequences could identify zoonotic viruses with reasonable accuracy [66]). Genome-based models are also inherently constrained by the constant evolution of new viral lineages [19,67]; while influenza sequence data are abundant enough to make protein-based models that are somewhat insensitive to this problem [61], other situations—such as the emergence of a novel recombinant canine coronavirus (alphacoronavirus 1) that can infect humans—are harder to anticipate [68]. Beyond genomic features, ecological data on viruses are often even more limited; for example, metadata on host range—a key predictor in many previous zoonotic risk studies—is even more underdeveloped, with 20–40% of associations missing in even the smallest, best-sampled networks [69]. A recent study showed that graph embeddings of the host–virus network could improve the performance of a genomic model of viral zoonotic potential—adding high-dimensional data to a model otherwise limited to a few hundred points—but using an imputed host–virus network even further improved predictions by overcoming gaps in viral host range data [70]. As these examples highlight, data limitations are likely to change as viruses are discovered at an increasing rate. Still, in the interim, experts will continue developing unique solutions to facing data sparsity that will advance both the basic biology and the computational tools of machine learning.

It is difficult to define what zoonotic risk technology might look like within the next 10 years. As models predicting zoonotic potential become more advanced, it may be increasingly possible to also address epidemic potential—one recent example was able to identify transmissible human viruses with greater than 80% accuracy [10], while others have nodded towards the possibility that mortality might also be predictable [29,31]—and to use machine learning more fully for comprehensive risk assessment. This nascent field of research is likely to grow exponentially as post-pandemic investments transform both the available data describing the global virome and the institutional support for modelling research and development (and associated training in higher education). Especially if this work focuses on improvement through validation and cross-talk between experimentalists and modellers, we anticipate that the predictive accuracy and reliability of these technologies will continue to grow. Previous work, especially from virologists, has been sceptical that these approaches might become a reliable source of inference [51,67]; however, prior work anticipating the level of predictive resolution that exists today has also historically been subjected to similar scepticism. Moving past these concerns may require a transition from primarily computational (sometimes mechanism-agnostic) models—which can perform well at any given task, but may be harder to interpret through different disciplinary lenses—and towards a deeper conceptual synthesis of virology and computational biology, focused on identifying the rules of life that underpin host–virus interactions through a computational lens. As models become powered by growing datasets cataloguing the global virome [2,71–75], and more complex microbiological predictors that capture more granular host–virus interactions, it is difficult to imagine today how accurate and valuable they might become. If their potential for global health manifests, we should prepare now to guard against potential misuse, including monopolization in high-income countries, and to anticipate important matters of equity, including the equitable sharing of the benefits arising from their use.

## 3. Connecting computational and empirical work

Zoonotic risk technology can suggest which viruses may have zoonotic potential, with a non-trivial degree of uncertainty, but further confirming that risk requires laboratory characterization. For example, successful viral replication in humans requires tens to hundreds of protein–protein interactions, which may not be predicted from viral sequence data alone and require laboratory characterization [76–78]. Conversely, one of the greatest strengths of these tools is their ability to narrow down the list of (potentially millions of) viruses for risk assessment procedures that require complicated, sometimes-expensive experiments. For example, experimental evaluation of host competency may require establishment of cell lines from new species [79] or non-model organism systems in the laboratory with a suite of associated challenges including unique housing requirements, low fecundity, a lack of commercial availability, few species-specific laboratory reagents and often scant baseline data upon which to support health evaluations. Focusing on establishing these systems for the wildlife viruses with the highest predicted risk could be a way to direct effort and minimize costs, particularly if model-to-validation steps are built in that can validate the underlying biological reasoning (e.g. predicting cell entry based on receptor sequences followed by high-throughput functional testing [80]). Conversely, experimental work will point to new needs in model development (e.g. when cell line experiments identify previously uncharacterized receptors, these can be incorporated back into the modelling process).

This aspect of zoonotic risk assessment can be complicated by concerns that this might require gain-of-function experiments, which use genetic editing or forced adaptation experiments to induce new phenotypes, potentially expanding the host range, pathogenesis or mode of transmission of a pathogen [81]. While these experiments have been critical to previous work—for example, by demonstrating the epidemic potential of highly pathogenic avian influenza through directed mutagenesis and serial passage to recover a virus capable of airborne transmission [82,83], or by demonstrating the potential of SARS-like viruses to jump from bat reservoirs into human populations [84]—they also face tremendous scrutiny, given potential or perceived biosafety and biosecurity risks, including those potentially arising from dual-use research of concern [85]. (Importantly, most host–virus interaction research—including *in vitro* and *in vivo* experimental infections—are not actually ‘gain-of-function’ experiments, but may also be mislabelled or misidentified as such by the public or media.) These concerns are likely to face even greater scrutiny given public conversations about SARS-CoV-2's as-yet-unknown origins and the emergence of unsubstantiated origin theories centred around biosecurity lapses [86].

There are a tremendous diversity of experimental approaches stopping short of gain-of-function experiments that can be used to validate predictive models and offer a more operationalized view of the problem. Among these are experimental infections to test the ability for cell entry and receptor usage, replication, pathogenesis, evasion of host immune responses, assembly and egress, and onward transmission [80,87]. While experimental infections of live animal models [88] or captive wildlife colonies are often critical to answer these questions, a number of *in vitro* approaches could also be used more extensively in partnership with model-driven work. *In vitro* laboratory models, including cell lines or organoids, may facilitate identification of host receptors required for viral entry or key immunological factors that influence viral replication in the reservoir host [89]. Replication incompetent pseudovirus particles and self-replicating, non-infectious viral replicon systems allow researchers to characterize specific host–virus molecular interactions in *in vitro* systems, or test the efficacy of therapeutic interventions, without the need for the elevated biocontainment measures that may accompany the use of the live virus. Each of these laboratory approaches can offer targeted methods to validate predictions from machine learning models, such as virus–human compatibility, ability for viral replication and productive infection, and disease pathology, tissue tropism or courses of infection. Many of these methods can be used without the requirement for high-containment laboratories, which is particularly important to ensure that a wide variety of different viral groups can be studied safely yet at scale and across country contexts.

Experimental work can further help identify the (modellable) molecular barriers to zoonotic emergence. Each stage in the viral life cycle represents an opportunity to improve model performance, but will require the gathering and reconciliation of data across multiple host–virus systems and experimental approaches. For example, laboratory experiments are likely to vastly improve model performance upstream by offering new kinds of predictor data that reflect the various types of host responses to infection. These may include broad comparative data on host transcriptomic or proteomic responses to infection [90], or host–virus protein–protein interactions [77], which may help identify the mechanisms of infection and pathogenesis in humans even when collected from animal model systems [90]. Through better collaboration among statistical modellers and empiricists, future development of zoonotic risk technologies can iteratively validate or falsify model predictions, helping to improve the accuracy and applicability of predictive models over time. While some modelling publications may, therefore, recommend further characterization of specific viruses, this will be unlikely to occur without active partnership between modellers and experimentalists, given that the priority is often placed on known and recurrent threats.

## 4. Theory to technology, technology to toolkit

Most zoonotic risk technology is developed with the stated intent to contribute positively to human health and reduce the future burden of emerging zoonotic viruses. However, the knowledge that a virus poses a threat to human health often exists for years, even decades, before a catastrophic outbreak [33]. Zoonotic risk technology may, therefore, have limited benefit to global health without careful, intentional work focused on application and actionability. The pipeline from technology development, to implementation, to risk mitigation is likely to only succeed at first in specific, narrow use cases.

First, and most foundationally, predictive modelling work will always be disconnected from global health if the endpoint is in the academic literature. This is particularly the case if research groups are separated geographically and practically from the direct impacts of potential spillover events, and choose not to pursue collaboration and knowledge exchange with local experts, limiting both the expertise available to properly design and contextualize work, and the channels available for possible dissemination and outreach. Similar challenges have been identified for related modelling problems, like the development and deployment of early warning systems or real-time epidemic forecasting [91,92]. Engaging practitioners, policymakers and stakeholders in the participatory design, release and ongoing improvement of infrastructure is likely to increase the value of zoonotic risk technology, as will designing open tools with public interfaces (open-source software or Websites, e.g. FluLeap: https://fluleap.bic.nus.edu.sg/) and based on open, interpretable data. These tools should be adaptable in order to more accurately reflect scientific advances, as well as changing user needs, over time. They must report appropriate, interpretable and locally relevant metrics of risk to inform decision-making, with uncertainty presented as transparently as possible, including where uncertainty comes from (e.g. data limitations versus model calibration) and how uncertainty correlates with the outcome variables. Scientists may also be called on to develop a new language for conveying context-dependent risk and communicating uncertainty to different audiences, including properly disclaiming results such that public, private or health sector responses neither over-react to high-risk predictions nor under-react to low-risk predictions. This enduring challenge extends into many other aspects of global health and disease ecology.

At least in the near term, it is unlikely that any specific, coordinated and effective response will be mobilized based solely on the identification of a novel virus with zoonotic potential. Resource scarcity prevents the development of individual surveillance systems or biomedical research-and-development programmes for each of the thousands of wildlife viruses with zoonotic potential; existing programmes focused on the narrowest set of expert-assessed high-risk threats (e.g. influenza A viruses, betacoronaviruses or henipaviruses) are already over-encumbered. These programmes may, however, benefit from technology that identifies zoonosis-relevant evolutionary shifts in viruses circulating in wildlife (e.g. the emergence of a Nipah virus lineage with greater estimated transmissibility, a long-standing concern in some biosecurity circles [93]).

More broadly, these tools may find applications in existing One Health surveillance programmes focused on high-risk interfaces between wildlife, domestic and captive animals, and humans. Zoonotic risk technology is likely to be most actionable at small scales: a local inventory of wildlife viruses can be ranked according to risk, frequency and degree of human–animal contact, with the highest-risk viruses incorporated back into local surveillance priorities. For studies reporting the discovery of novel animal viruses, this step can be simple as an additional analysis, with at least one published example of this use case [94]. However, if studies are designed with these kinds of assessments in mind, they may also be able to collect additional data with tremendous value. For example, sequence-based viral discovery often focuses only on viral reads and discards data from the host [95]. The host-derived sequence data contain crucial information about the host response that could provide insight into a given virus' pathogenic potential in an animal or human host, allowing for further surveillance prioritization of both host and virus species. The use of these studies is also limited by the quality of data shared in the public domain: sharing of standardized and validated data such as host species identification, location, specimen type and date of collection in a centralised resource, rather than lost in a publication (if at all), is one of the first steps to a truly collaborative approach.

Once high-risk viruses are identified and reported in wildlife monitoring studies, these pathogens may also be identified and flagged earlier in samples collected by programmes that passively monitor the health of high-risk human populations (e.g. livestock keepers or wildlife traders) and sentinel hosts like livestock, or actively screen human populations for novel pathogens by investigating undiagnosed febrile illnesses [96–99]. Behavioural change or occupational safety interventions may then be targeted to reduce spillover risk for high-risk human populations, though they may be most feasible or successful if they target risky exposure to specific host species with multiple high-risk viruses and frequent human contact (especially if they already match local priorities), thereby protecting against their entire *zoonotic virome*. For example, while Nipah virus is the highest-priority zoonotic threat hosted by the Indian fruit bat (*Pteropus medius*), interventions that reduce Nipah exposure in humans may also protect against the other 50+ viruses that these bats host [100]. Similarly, the reservoir for Lassa virus carries a number of other bacterial zoonoses, and rodent control can reduce transmission risk across this range of threats [101]. While many of these reservoirs are known today from their role in pathogen spillover, a number of other high-risk species are presumably unknown; building on previous work that characterizes zoonotic risk using ecological traits correlated with ‘hyperreservoirs’ [25], future research may include characterizing these species' viromes, and estimating the risk that they pose in aggregate.

## 5. Failure to equitably share benefits may limit impact

As the failure to ensure global access to diagnostics, therapeutics and vaccines during the COVID-19 pandemic has demonstrated [102], the distribution of the benefits from health technologies, particularly novel technologies, is inequitable and a global injustice. Global health must not simply aspire to principles of health equity and social justice, but must also make equitable access to life-saving technologies a condition precedent to their development and use. This must be a priority in the development and use of zoonotic risk technology, which may also pose a unique set of problems for both researchers and practitioners. These technologies depend on open data sharing, both to create sufficient training sets for artificial intelligence and to actually apply them for risk assessment and subsequent mitigation. Community efforts to share human and animal sequence data at sufficient scales (i.e. to generate feature sets for advanced machine learning) exist for just a handful of high-profile viruses, nearly exclusively as part of international coordination on pandemic preparedness and response (e.g. influenza A and SARS-CoV-2 data sharing via the GISAID platform), while all-purpose repositories like GenBank still only capture a fraction of known viruses (as many are bottlenecked by taxonomic ratification), and lack essential metadata needed for prediction. Both face challenges with regard to contributors receiving credit and attribution for research (especially modelling studies) based on the data they submit (box 1).

Box 1. **Crediting researchers for re-used, open sequence data.**

When ‘big data’ becomes available at scales that allow machine learning (or other intensive secondary analysis), the researchers who compiled the data often receive exponentially diminishing credit through academic incentives. Existing public data repositories, including GISAID and NCBI GenBank, have no indexable source attribution for sequence data. GISAID requires acknowledgement of the source, but such acknowledgement is not a trackable metric contributing to career development; similarly, GenBank accession numbers assist in the reproducibility of analyses, but are not indexed, and cannot be easily tracked by contributors as a career metric. This can disincentivize open data sharing if it is seen as a ‘non-promotable’ task for those generating the data, given that other indexed metrics like citations may be used to determine scientific impact when evaluating funding proposals or in hiring and promotion decisions.

Moreover, this system currently benefits users of public data repositories more than those who generate the data. In several instances during the COVID-19 pandemic, laboratories generating SARS-CoV-2 sequence data have been stretched thin with pandemic response and were unable to annotate, analyse and publish on their data before computational or academic laboratories used the data in their own publications. Similar practices are particularly divisive when researchers use data generated by public health laboratories in developing nations without co-authorship, collaboration or indexed citation of the source.

One potential solution would be an indexed DOI for sequence data; similar approaches are used for aggregate data in biodiversity research (e.g. by the Global Biodiversity Informatics Facility; gbif.org), and while many studies fail to follow recommendations for proper attribution, these procedures are a reasonable first step for fair credit.

These problems become more complex with regard to the deployment of zoonotic risk technology itself. Initially, there may be resistance to using these tools: scientists who gather novel sequence data may rightfully be hesitant to upload unpublished data to online Web tools for zoonotic risk prediction without clear and enforceable protections against data reuse by the curators of such tools, even if these tools are curated by a trusted third-party (though access to this technology may inherently change power dynamics). This is only one concern out of a broader set of issues around *access and benefit sharing* for viral surveillance. Based in countries’ sovereign rights to determine the use of resources within their territory, access and benefit-sharing regimes seek to redress and prevent injustices arising from the exploitation of genetic resources, and from the inequitable sharing of the benefits that arise from their use. Some protections and norms around the sharing of physical pathogen samples and the benefits arising from their use are reflected under the Nagoya Protocol on Access to Genetic Resources and the Fair and Equitable Sharing of Benefits Arising from their Utilization (Nagoya Protocol) to the Convention on Biological Diversity. Under the Nagoya Protocol, countries may implement domestic legislation that requires foreign researchers seeking access to pathogen samples, or in some cases even data related to those samples, to obtain the country's prior informed consent and the conclusion of mutually agreed terms that include benefit sharing, such as attribution in publications, capacity building, technology transfer or intellectual property rights. Depending on the terms agreed, the use of genetic sequence data derived from pathogen samples may be restricted, including both preventing the sharing of sequence data in open access databases, requiring open sharing or the sharing of any diagnostic tools developed using the sequence data.

Given that a growing number of laboratories are readily able to synthesize viruses from their genome sequences (e.g. horsepox [103] and SARS-CoV-2 [104]), there are concerns that the bargain underpinning access and benefit-sharing regimes provided by physical samples—like those established in the Nagoya Protocol—may be in flux. If zoonotic risk technologies allow researchers to identify high-risk viruses with reasonable certainty before laboratory characterization, this could add an additional layer of complexity. Sequence sharing and synthesis of SARS-CoV-2, in addition to the global failure to equitably distribute associated vaccines, diagnostics and therapeutics, may motivate attempts to expressly address these gaps in international legal instruments. These could include, for example, potential revision to the International Health Regulations (2005) or the Nagoya Protocol, or new international law, such as a Pandemic Treaty. Any international governance reform should actively consider the importance, on equal footing, of open data sharing and the equitable sharing of the benefits of novel technologies like zoonotic risk technology.

Even if zoonotic risk technologies are easily applied without challenges around sequence data sharing, there may be gaps between intentions and actionable science. When high-risk viruses are identified, findings may be kept private until they are published much later in peer-reviewed journals, both to protect credit for scientific discoveries and to accommodate governments' hesitancy to release information that could create fear or stigmatization. This could reinforce the disconnect between viral sampling and actionable science for global public health. At present, announcements about the discovery of notable animal viruses are often made ad hoc either by press release or conventional publishing methods. If zoonotic risk technology becomes a widely adopted part of surveillance, new governance processes will probably need to be developed that protect researchers’ careers and credit, but also ensure that announcements are transparent and verifiable, particularly if alarming or unusual results (e.g. the hypothetical discovery of a filovirus with zoonotic potential in bats in the USA) are likely to motivate public or international concern.

Another set of issues could arise around who benefits from zoonotic risk technology. It seems plausible that these technologies might mostly benefit from the research effort and data sharing occurring in tropical countries, where zoonotic viral diversity is believed to be highest [11]. However, their development might mostly further the careers of researchers in high-income countries in North America and Europe, particularly if developed by experts who are unattuned to power dynamics in global health. Equally concerning, we identify a possibility that these tools will largely be developed as proprietary ‘risk assessment algorithms' by corporate ‘data science for impact’ programmes, for-profit global health firms and non-profit organizations, just as they have been for the development of pandemic insurance programmes or similar analytics. In these circumstances, and without appropriate governance, the countries with the highest burden of zoonotic emergence might find their own data (repackaged in an analytic format) sold back to them at a premium by scientists and corporations from high-income countries. Open sharing of academic research could help scientists undermine this trend and provide tools directly to end users in public health, or assist them in developing their own tools, but may simply accelerate advances in zoonotic risk technology without changing the existing colonial framework of global health. Involving researchers from low- and middle-income countries—and supporting their leadership in this field (particularly, to a greater extent, in future workshops on this topic, which could advance this issue further than the present workshop did)—will help limit these shortfalls. This can be particularly facilitated by using virtual workshops (with attention to different accessibility challenges) and by supporting in-person workshops around the world, and by coordinating participation proactively through existing zoonotic disease and laboratory networks (e.g. SEAOHUN, OHCEA or the CREID centre network). Involving researchers from the discipline of science and technology studies may also lead to a more honest and critical appraisal of the ethical issues surrounding the emerging technology, and who it benefits or harms.

Finally, we anticipate that zoonotic risk technology may replicate existing, and potentially create new, ethics and governance problems in *synthetic biology*. Just as convolutional neural networks and other kinds of artificial intelligence can be used to fabricate realistic images entirely through predictive algorithms (e.g. thispersondoesnotexist.com), zoonotic risk technology might be used to generate novel viral sequences (and potentially synthetic viruses) with high predicted zoonotic and epidemic potential. Already, researchers have used these approaches to simulate alternate coronavirus spike protein sequences that might be able to infect human cells [105]. These approaches might support biomedical work; for example, synthetic spike proteins could be used to test a candidate universal betacoronavirus vaccine for its value across ‘unsampled evolutionary space’. However, if biomedical companies attempt to patent these sequences, they could create new problems for future sample sharing, therapeutic and vaccine development, or outbreak response if viruses with the relevant sequence someday emerge—potentially at the expense of some countries more than others. While similar issues have been raised before during zoonotic outbreaks [106], the novelty of *simulated zoonoses* might create new complications for intellectual property law. Moreover, viral ranking algorithms or artificially simulated virus sequences might also be used by a malicious actor, highlighting the need to involve scholarship from the ‘dual use’ field of bioethics.

## 6. Prediction is not prevention

Zoonotic risk technology may become an asset in the emerging disease toolkit, but overselling this technology or understating uncertainty will lead to preventable divergences between expectations and scientific possibility. Models may ultimately have profound clinical and field applications, but the uncertainty around risk estimates and likelihood of inaccurate predictions must be carefully communicated. As part of that, epistemic differences in disciplinary conceptions of uncertainty may need to be bridged: for example, a model may make ‘errors’ simply because reality is a stochastic observation of underlying risk landscapes, and a technology that correctly infers probabilities or risk landscapes will still never perfectly represent reality. (These may play into other disciplinary tensions about what ‘prediction’ means: to public health experts and the public, prediction is often synonymous with anticipating future events, but to computational biologists, it may more often be used to describe accurate inference about biological possibility.) Further, there is no substitute for experimental work, and bench virology will play a critical role to generate the necessary data for model development and validation. Zoonotic risk technology is also no substitute for general public health preparedness; even though these tools could be used in the future to estimate the risk posed by newly discovered viruses as soon as the first genome becomes available, many viruses are still likely to continue to enter human populations before they have been characterized in animals. Whether these outbreaks become epidemics or pandemics is a problem outside the scope of the technologies we discuss.

Therefore, we warn that investments in research and development on topics like machine learning or animal virus genomics must not come at the expense of other essential kinds of modelling work (e.g. work focused on virus transmission and spread, or identifying the most consequential surveillance gaps), or more importantly, at the expense of non-technological investments in health systems strengthening, including attainment of universal health coverage, and similar aspects of pandemic preparedness. Similarly, it is possible that interest in pre-emergence zoonotic viruses might conflict with, redirect, or undermine local priorities like water and food-borne diseases (and sanitation), agricultural, high-burden communicable diseases (e.g. HIV-AIDS, tuberculosis and malaria) or non-communicable diseases; interventions may even disrupt local interests and norms, potentially weakening outbreak response during emergencies. If the post-pandemic period becomes dominated by this narrow subset of research priorities, researchers will need to be individually careful in order to accurately and fairly present the value and importance of their work (an imperative that will be encouraged by efforts to reduce funding scarcity in this space).

At the same time, it is indisputable that zoonotic risk technology is currently limited in both development and application by data scarcity, and that the only solution to this is continued or greater investment in data collection—particularly in basic science. Post-pandemic investment in coordinated programmes for viral discovery, One Health surveillance, bench virology and other kinds of laboratory capacity are all likely to generate vital data that can improve the performance of these technologies, and remedy critical gaps in our current understanding of the global virome. These will be most effective if investments are maximized in the hotspots of zoonotic emergence, if modellers are engaged in the process to support data collection and processing in reusable formats, and—perhaps most importantly—if these investments are made with the aim of improving outbreak prevention and preparedness entirely independent of the success or failure of zoonotic risk prediction as a scientific outcome.

Finally, we suggest that ongoing work is required to benchmark the accuracy and value of these technologies, that transparency and uncertainty be key facets of their presentation and most importantly that the scientific community remains prepared for ‘surprises’. (In a strikingly timely example, only days before the submission of this manuscript, the first-ever report of human infections with H5N8 avian influenza A virus was released—a strain that was, surprisingly enough, able to be correctly identified as human by a previously published model that had never encountered a zoonotic H5N8 virus in the training data [107].) Models are only as powerful as the data that inform them, and with such a small percentage of the global virome described to date—and new viruses evolving constantly—it seems likely that the next generation of risk prediction systems, and public health infrastructure that may come to rely on them, will face a number of entirely unexpected threats.

## 7. Conclusion

At present, efforts to predict ‘Disease X’—an unknown threat that could someday reach humans—rely on a mix of expert opinion and laboratory virology. In the coming years, researchers may increasingly be able to rely on statistical inference and artificial intelligence as another line of evidence. The availability of these tools might make wildlife disease surveillance programmes more impactful, and better connect their work to outbreak prevention, but only if the many barriers to actionable science are identified and addressed proactively. The first and most foundational step is building a global community of open science that pursues collaborative, interdisciplinary and impactful work at the nexus of virology, computational biology and global health. Building that community will have benefits far beyond the narrow problem of zoonotic risk technology, and will make the world more prepared for any possible future threat.

## Data accessibility

This article has no additional data.
